# A comprehensive review of hydroxyurea for β-haemoglobinopathies: the role revisited during COVID-19 pandemic

**DOI:** 10.1186/s13023-021-01757-w

**Published:** 2021-03-01

**Authors:** Nirmani Yasara, Anuja Premawardhena, Sachith Mettananda

**Affiliations:** 1 Department of Paediatrics, Faculty of Medicine, University of Kelaniya, Thalagolla Road, Ragama, 11010 Sri Lanka; 2 Department of Medicine, Faculty of Medicine, University of Kelaniya, Ragama, Sri Lanka; 3 Colombo North Teaching Hospital, Ragama, Sri Lanka

**Keywords:** Hydroxyurea, Thalassaemia, Sickle cell disease, Haemoglobinopathies, γ-Globin induction, Blood transfusion, Ribonucleoside diphosphate reductase, COVID-19

## Abstract

**Background:**

Hydroxyurea is one of the earliest drugs that showed promise in the management of haemoglobinopathies that include β-thalassaemia and sickle cell disease. Despite this, many aspects of hydroxyurea are either unknown or understudied; specifically, its usefulness in β-thalassaemia major and haemoglobin E β-thalassaemia is unclear. However, during COVID-19 pandemic, it has become a valuable adjunct to transfusion therapy in patients with β-haemoglobinopathies. In this review, we aim to explore the available in vitro and in vivo mechanistic data and the clinical utility of hydroxyurea in β-haemoglobinopathies with a special emphasis on its usefulness during the COVID-19 pandemic.

**Main body:**

Hydroxyurea is an S-phase-specific drug that reversibly inhibits ribonucleoside diphosphate reductase enzyme which catalyses an essential step in the DNA biosynthesis. In human erythroid cells, it induces the expression of γ-globin, a fetal globin gene that is suppressed after birth. Through several molecular pathways described in this review, hydroxyurea exerts many favourable effects on the haemoglobin content, red blood cell indices, ineffective erythropoiesis, and blood rheology in patients with β-haemoglobinopathies. Currently, it is recommended for sickle cell disease and non-transfusion dependent β-thalassaemia. A number of clinical trials are ongoing to evaluate its usefulness in transfusion dependent β-thalassaemia. During the COVID-19 pandemic, it was widely used as an adjunct to transfusion therapy due to limitations in the availability of blood and logistical disturbances. Thus, it has become clear that hydroxyurea could play a remarkable role in reducing transfusion requirements of patients with haemoglobinopathies, especially when donor blood is a limited resource.

**Conclusion:**

Hydroxyurea is a well-tolerated oral drug which has been in use for many decades. Through its actions of reversible inhibition of ribonucleoside diphosphate reductase enzyme and fetal haemoglobin induction, it exerts many favourable effects on patients with β-haemoglobinopathies. It is currently approved for the treatment of sickle cell disease and non-transfusion dependent β-thalassaemia. Also, there are various observations to suggest that hydroxyurea is an important adjunct in the treatment of transfusion dependent β-thalassaemia which should be confirmed by randomised clinical trials.

## Background

Hydroxyurea is an antimetabolite drug which is in use for over four decades [1]. It is one of the earliest drugs that showed promise in the management of haemoglobinopathies and is the first FDA approved drug for the treatment of sickle cell disease (SCD) [2]. Despite being in use for several years, many aspects of hydroxyurea are either unknown or understudied; specifically, its usefulness in β-thalassaemia major and haemoglobin E β-thalassaemia is unclear [3]. However, with the recent COVID-19 pandemic, it is increasingly used in the management of β-haemoglobinopathies. In this review, we aim to explore the available in vitro and in vivo mechanistic data and the clinical utility of hydroxyurea in β-haemoglobinopathies with a special emphasis on its usefulness during the COVID-19 pandemic.

### β-Haemoglobinopathies

Haemoglobinopathies are a group of heterogeneous disorders which are due to abnormalities of human globin chains that form adult haemoglobin (Hb), HbA. HbA is a tetramer of two α- and two β-globin chains (α_2_β_2_) which are encoded by α- or β-globin genes located in chromosome 16 and 11 respectively [4]. The genetic mutations of β-globin gene result in β-haemoglobinopathies, of which the most common are SCD and β-thalassaemia.

SCD is due to a recessively inherited point mutation that leads to the substitution of adenine to thymine at 6^th^ codon of the β-globin gene (β^S^ allele) [5]. This change replaces glutamic acid with valine at position six of the β‑globin chain generating an abnormal β-globin (HBB Glu6Val). These mutated β-globin chains when combined with normal α-globin chains form structurally abnormal haemoglobin, HbS, which polymerises in deoxygenated states to cause sickling of red blood cells (RBCs). Patients who are homozygous for β^S^ allele develop the most severe disease, sickle cell anaemia (SCA). Compound heterozygosity of HbS and HbC causes haemoglobin SC disease whereas that of HbS and β-thalassaemia results in sickle β-thalassaemia [5].

In contrast to SCD, β-thalassaemia is caused by quantitative reduction in the synthesis of β-globin chains due to mutations in and around the β-globin gene [6]. Inheritance of these mutations in homozygosity often results in β-thalassaemia major while heterozygous states lead to β-thalassaemia trait. Additionally, a point mutation at codon 26 of the β-globin gene results in structurally abnormal β-globin protein (β^E^) which is synthesised at reduced rates to give rise to the phenotype of β-thalassaemia. The haemoglobin molecule that is composed of α- and β^E^-globin chains, is known as HbE (α_2_β^E^_2_). The compound heterozygous state of β-thalassaemia and β^E^ mutation is known as HbE β-thalassaemia.

Although β-haemoglobinopathies are among the first diseases to be characterised precisely at the molecular level, its management is mostly limited to supportive treatment [7]. The supportive treatment of SCD includes avoidance of precipitants, general treatment of vaso-occlusive crises and prevention of organ-specific complications [8]. Conversely, regular blood transfusions and long-term iron chelator medication form the cornerstone of supportive treatment of β-thalassaemia. With the advent of novel genetic-based approaches to treat human diseases, several experimental therapies are on development for SCD and β-thalassaemia [9–11]. For example, silencing of human α-globin by mutating α-globin enhances and allelic disruption of aberrant splice sites in specific β-thalassaemia mutations using genome editing have been successful in pre-clinical studies [12, 13]. Furthermore, upregulation of fetal haemoglobin (HbF) by de-repressing γ-globin is a well-established pathway that is being utilised to devise a cure for β-thalassaemia [14, 15]. Hydroxyurea is one of the medications that has shown promise in achieving this.

### Pharmacodynamics of hydroxyurea

Hydroxyurea (also known as hydroxycarbamide) is an antimetabolite S-phase-specific drug that reversibly inhibits ribonucleoside diphosphate reductase (rNDP) enzyme [16]. This enzyme catalyses the conversion of ribonucleotides to deoxyribonucleotides which is an essential step in DNA biosynthesis. Inhibition of rNDP and impaired synthesis of DNA prevent the progression of cells from the G1 or pre-DNA synthesis phase of the cell cycle. Also, hydroxyurea is cytotoxic to S-stage cells resulting in their destruction. The enzyme inhibitory effect of hydroxyurea is limited to the de novo synthesis of DNA and DNA repair; it does not have an effect on RNA or protein synthesis [17, 18].

In addition, hydroxyurea is also known to induce γ-globin in human erythroid cells. γ-Globin is the predominant type of β-like globin expressed during fetal life which is gradually suppressed after birth to minimal levels by the end of the first year of life. In patients with β-haemoglobinopathies, due to the absence of normal β-globin chains, any increase in γ-globin becomes useful [13]. This is because γ-globin chains can combine with α-globin to form HbF. Therefore, the ability to induce γ-globin is the most important action of hydroxyurea in β-haemoglobinopathies [19].

### Pharmacokinetics of hydroxyurea

Oral hydroxyurea is easily absorbed through the gastrointestinal tract and distributed rapidly and widely in the body. The peak plasma concentration is reached 1 to 4 h after an oral dose. It is recommended to start with a lower dose of 10–15 mg/kg/day and gradually increase in steps of 2.5–5 mg/kg/day to a usual dose of 15–30 mg/kg/day (the maximum dose is 35 mg/kg/day). The effects of hydroxyurea are transient as the drug is rapidly cleared from the circulation. Elimination of hydroxyurea is mainly through urine after being metabolised in the liver [1, 20, 21].

### Effects of hydroxyurea on haematology, erythropoiesis and haemorheology

#### Improvement in haematological parameters

**Fig. 1:**
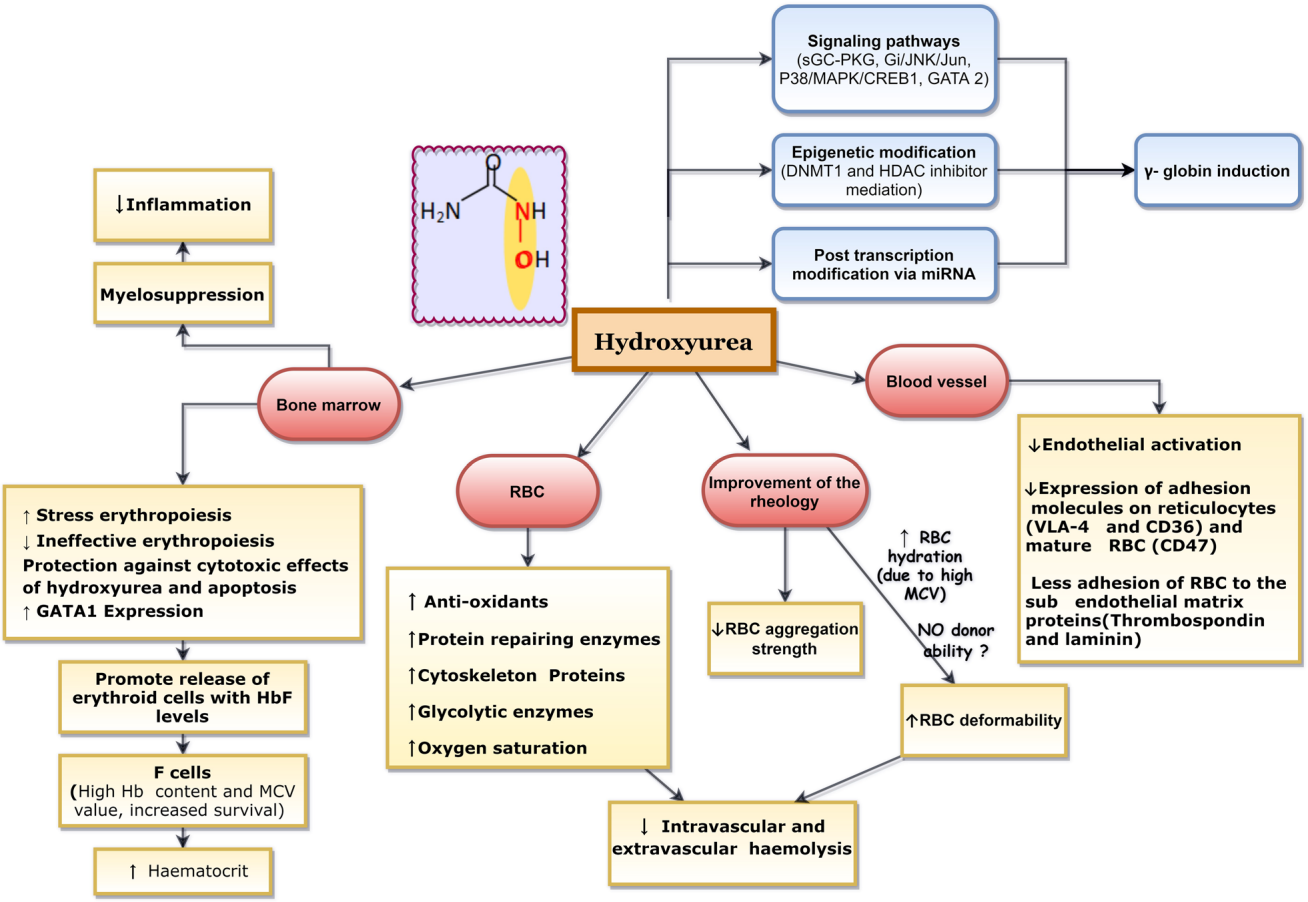
Effects of hydroxyurea in haemoglobinopathies. Hydroxyurea exerts favourable changes in red blood cells, vascular endothelium, and bone marrow in patients with β-haemoglobinopathies through a number of cellular and molecular pathways. Abbreviations: RBC, Red blood cells; Hb, Haemoglobin; sGC, Soluble guanylate cyclase; PKG, cGMP-dependent protein kinase; Gi/JNK/Jun, G(i)-dependent activation of c-Jun N-terminal kinase; MAPK-p38, mitogen-activated protein kinase; CREB1, cAMP response element-binding protein 1; DNMT, DNA methyltransferase; HDAC, Histone deacetylase

The primary mechanism of action of hydroxyurea in β-haemoglobinopathy is upregulation of γ-globin gene expression in erythroid cells. Subsequently, the γ-globin chains can combine with α-globin in RBCs to form HbF (α_2_γ_2_) [22]. HbF shows heterogeneous distribution among RBCs of adults with only a small proportion of cells showing detectable levels. The cells with a higher proportion of HbF (approximately 20–25%) are known as F cells, and they have the ability to escape from deleterious effects of abnormal haemoglobin found in β-haemoglobinopathies. Hydroxyurea has shown to enhance the F cell percentage in the circulations through several mechanisms (Fig. 1). Thereby it has shown to improve haemoglobin level, haematocrit, mean corpuscular volume (MCV) and mean corpuscular haemoglobin (MCH) in patients with β-haemoglobinopathies [23–27].

#### Inhibition of ineffective erythropoiesis

Ineffective erythropoiesis is one of the main contributing factors for anaemia in β-haemoglobinopathies. Several studies have shown that hydroxyurea inhibits ineffective erythropoiesis in these patients. Some studies have demonstrated a decrease in nucleated RBCs, while others have shown a reduction in soluble transferrin receptor levels, both of which are indirect indicators of ineffective erythropoiesis [28, 29]. In addition, hydroxyurea causes intermittent suppression of erythroid progenitors due to its cytotoxicity and stimulates cell stress signalling. Induction of cell stress signalling promotes the release of erythroid progenitors containing high HbF [30, 31].

#### Improvement in haemorheology

Haemorheology is the study of flow and biophysical properties of blood which is predominantly determined by the size of blood vessels and the viscosity of blood. Blood viscosity depends on several factors that include haematocrit, plasma viscosity, RBC deformability and RBC aggregation-disaggregation properties [32]. Increasing haematocrit, MCV of RBCs and plasma viscosity increase blood viscosity whereas, improved cell hydration and deformability decrease viscosity [33]. RBC deformability depends on several factors that include cytosolic viscosity (determined by mean corpuscular haemoglobin concentration), membrane viscoelasticity (dependent on cytoskeleton proteins and lipid bilayer properties) and surface area to volume ratio.

Abnormal haemorheology is one of the predominant pathophysiological features of SCD. SCD is characterised by mechanically fragile abnormal RBCs with limited deformability which is more pronounced under hypoxic conditions. Hydroxyurea improves the clinical severity of haemoglobinopathies by influencing the haemorheology. The increased haematocrit and haemoglobin level associated with hydroxyurea, do not have a significant influence on the blood viscosity, as the effects are compensated by the improvement in the RBC deformability and reduction in RBC aggregation strength. The improvement of the RBC hydration with hydroxyurea due to the increment in MCV is identified as one of the mechanisms causing improved RBC deformability. Some evidence suggests that the S-nitrosylation of β-spectrin from nitric oxide (NO) component of hydroxyurea also has an impact on the enhanced deformability [34, 35]. However, the effects of hydroxyurea on haemorheology requires further evaluation [33, 35, 36].

#### Bone marrow suppression

Hydroxyurea is a cytotoxic drug which was initially prescribed for malignancies. As the main mechanism of action of hydroxyurea is inhibition of rNDP enzyme, thus arresting cells at G1 or S phase of the cell cycle, bone marrow suppression is commonly associated with its treatment. Pancytopenia, leukopenia, neutropenia and thrombocytopenia are reported in numerous clinical trials [21, 37–40].

### Cellular and molecular effects of hydroxyurea

Hydroxyurea induces stress erythropoiesis by inhibiting the synthesis of DNA, thus giving a selective advantage to the expansion of the F cell population over the rapidly dividing HbA producing erythroid progenitors (Fig. 1). In addition, downregulation of genes responsible for chromosome organisation, translation and ribosome assembly has been observed in early reticulocytes following hydroxyurea treatment [41].

#### Effects on transcription factors

Hydroxyurea exerts a bi-modal effect on erythropoiesis in a dose-dependent manner by downregulating the expression of *GATA1* and upregulating *GATA2*. Both GATA1 and GATA2 are key transcription factors that regulate the proliferation and differentiation of erythrocytes. The changes in the expression of *GATA1* and *GATA2* favours the haemoglobin balance towards HbF by delaying RBC maturation and stimulating γ-globin expression [42]. Also, BCL11A, an important transcription factor that is responsible for postnatal silencing of γ-globin, is repressed by hydroxyurea promoting reactivation of γ-globin and induction of HbF [41, 43].

#### Effects on epigenetics

Several epigenetic mechanisms are responsible for the action of hydroxyurea in RBCs. Chromatin remodelling by nucleosome remodelling and deacetylase complex (NuRD) and methylation of CpG islands of DNA at the proximal γ-globin promoter by DNA methyltransferase 1 (DNMT1) is involved in hydroxyurea response. The main signalling pathways that are recognised to mediate the γ-globin induction effect of hydroxyurea are sGC-PKG, Gi/JNK/Jun and P38/MAPK/CREB1 [44–47]. The evidence supporting these pathways have been evaluated comprehensively in vitro using K562 cells and human erythroblasts.

#### Effects on microRNA

**Fig. 2:**
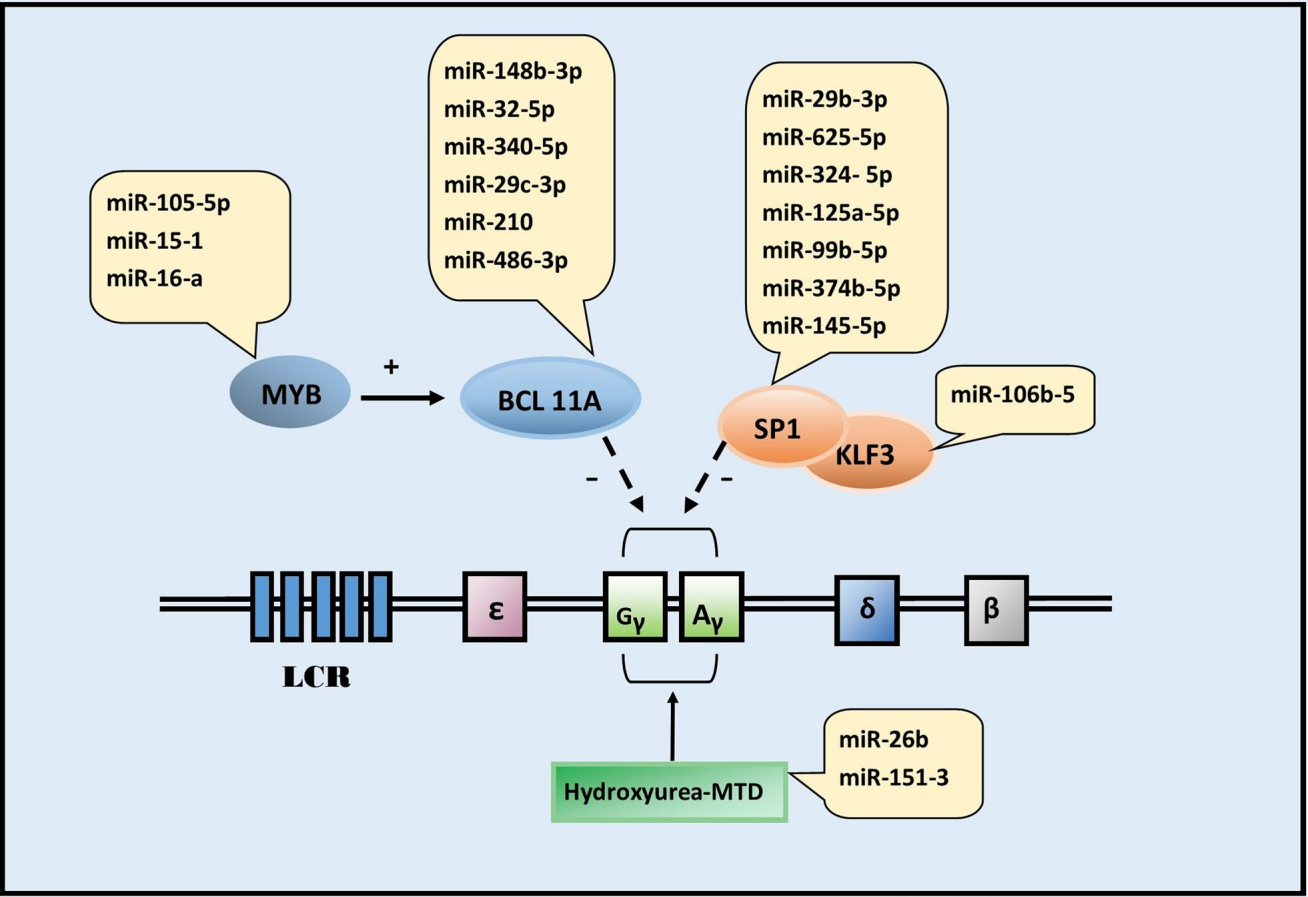
MicroRNA pathways involved in fetal haemoglobin induction effect of hydroxyurea. Human β-globin cluster consists of a locus control region (LCR) and four function genes (ε,γ,δ and β). γ-Globin is active during fetal life and is suppressed in adults by the action of several transcription factors that include Specificity protein 1 (SP1), Krüppel-like factor 3 (KLF3), Myeloblastosis oncogene (MYB) and B-cell lymphoma/leukaemia 11A (BCL11A). Hydroxyurea alters the expression of several microRNAs (miR) which act on these molecular targets to induce fetal haemoglobin. miR-26b and miR-151–3 stimulate γ-globin at the maximum tolerated dose (MTD) of hydroxyurea

Another novel pathway that was recently discovered to mediate the γ-globin gene expression by hydroxyurea is microRNA (miRNA) mediated post transcriptional regulation (Fig. 2). This is supported by evidence from studies done on both SCD and β-thalassaemia patients, and several miRNAs modulate fetal haemoglobin levels by targeting specific transcription factors associated with γ-globin expression [42, 48, 49]. Mnika and others discovered changes in the expression of 22 miRNA (out of 298 tested) following hydroxyurea treatment. The molecular targets of these differentially expressed miRNA are regulatory genes of γ-globin, which play an important role in fetal haemoglobin silencing in adults. Some miRNAs are associated with the maximum tolerated dose of hydroxyurea [50, 51]. Another study reported high levels of miR-210 and miR-486-3p in responders of hydroxyurea, strengthening the role of miRNA mediated regulation of HbF induction [49].

#### Effects on proteomics

The differences in protein expression in patients with SCD compared to healthy individuals and the influence of hydroxyurea on proteomics were uncovered in several studies [52–57]. RBC proteins that show significant changes following hydroxyurea treatment include antioxidants, oxidoreductases, structural membrane proteins, proteins responsible for protein degradation and repair, carbonic anhydrases, and glycolytic enzymes (Table 1). The major evidence on proteomics of hydroxyurea in SCD comes from a study done by Ragg et al. This study showed that 71 proteins were differentially expressed in children with SCD compared to healthy controls; however, the number reduced to 56 after treatment with hydroxyurea, suggesting beneficial effects of hydroxyurea in proteomics of these patients [52]. When the RBC membrane proteome was exposed to hydroxyurea in vitro, ten proteins were significantly upregulated. These include antioxidants (catalase and thioredoxin peroxidase), structural proteins, proteins required for protein repair (chaperonin containing TCP1 subunits) and carbonic anhydrase. A similar experiment performed in vivo among five patients with SCD revealed differential expression in cytoskeletal proteins following hydroxyurea treatment. Proteins like ankyrin, protein 4.1 and p55 which are important for maintaining the flexibility and the biconcave shape of the RBCs and few glycolytic enzymes involved in energy pathways were among them. Importantly, p55 levels were increased both in vivo and in vitro following exposure to hydroxyurea [54, 55, 57].

**Table 1:** Red blood cell proteins influenced by hydroxyurea

Protein category	Proteins
Antioxidants	Catalase Thioredoxin peroxidase Flavin reductase Peroxiredoxin-2 isoform
Oxidoreductase	Aldehyde dehydrogenase
Protein repair and degradation	Chaperonin containing TCP1 subunits Proteasome
Membrane structure	p55
Cytoskeletal	Anion exchanger band 3 Actin Stomatin Ankyrin Protein 4.1 Tropomodulin
Glycolytic enzymes	Glyceraldehydes-3-phosphate dehydrogenase Fructose-bisphosphate aldolase
Reticulocyte membrane receptors	Alpha 4 beta 1-integrin CD36
Mature RBC membrane receptors	CD47 CD147
Other	Carbonic anhydrase

Oxidative stress is one of the main underlying causes of disease pathology of SCD. In normal RBCs, band 3 protein regulates glycolysis; however, in sickled RBCs, this mechanism is disrupted due to hypoxia, impairing the antioxidant defence system [58]. Hence, induction of antioxidants is highly beneficial in protecting cells from the deleterious effects caused by the oxidative stress in SCD. Another evidence for the improvement of SCD by hydroxyurea is the reduction of lactate dehydrogenase (LDH) and arginase 1. LDH is an indicator of intravascular haemolysis, and arginase 1 is abundant in reticulocytes. The levels of both proteins are high in SCD due to increased haemolysis [59, 60] [61].

The transcriptomic studies on reticulocytes in patients with SCD demonstrated downregulation of low-affinity adhesion molecules after hydroxyurea therapy [54, 56, 62]. This finding emphasises another clinical advantage of hydroxyurea as adhesion of erythrocyte is the main underlying cause for complications associated with SCD. This response of hydroxyurea is supported further by in vitro studies which demonstrated a sustainable early reduction in adhesion to the subendothelial matrix proteins, thrombospondin and laminin, of sickled RBCs following hydroxyurea treatment [63].

Quantitative proteomics analysis in a cohort of patients with β-thalassaemia following treatment with hydroxyurea revealed that 28 proteins were differentially expressed after hydroxyurea treatment. The highest downregulation was observed in transferrin receptor protein-1 while significant upregulations were observed in haptoglobin, haptoglobins like protein and hemopexin. Haptoglobin and hemopexin are considered as scavengers of free haemoglobin and thus are sensitive markers of haemolysis and erythropoietic activity [53, 64–66]. In haemolytic diseases and haemoglobinopathies, serum levels of hemopexin and haptoglobin are significantly reduced. Therefore, the action of hydroxyurea in reducing haemolysis and improving erythropoietic activity in the bone marrow is compatible with the findings of high levels of hemopexin and haptoglobin after treatment. Additionally, the beneficial effects of hydroxyurea in ineffective erythropoiesis were further demonstrated by the reduction of transferrin receptor protein-1 observed in this study.

### Predictors of response to hydroxyurea

Many studies of hydroxyurea done on patients show a variable response to treatment. Several possibilities that explain this difference have been described recently. Differential susceptibility model delineates the ability of erythrocytes of responders and non-responders to act differently to hydroxyurea either in the induction of γ-globin pathway or of the cytotoxic effect of hydroxyurea. This is supported by studies that show the induction of HbF and F cells only in responders. For example, a study done by Colah and others showed 63% and 27% increase in F cell population in vivo and in vitro respectively in responder, however, did not show significant increases in F cell or γ-globin mRNA expression in non-responders [67].

Another theory for the variable response to hydroxyurea is known as the differential baseline model. This model proposes that hydroxyurea upregulates HbF in all patients, yet a significant increment is only visible in responders because they had a higher baseline HbF level (> 20%) compared to non-responders [68, 69].

Additionally, the differential response of responders and non-responders towards cell stress signalling pathways are supported by several experiments. A gene expression microarray study demonstrated upregulation of numerous genes which are associated with a protective role towards apoptosis and cell stress in erythroid progenitor cells (e.g. *ARG1* and *ARG2*) of responders giving them an immunity towards the cytotoxic effects of hydroxyurea. These genes had high expression before initiating hydroxyurea treatment in responders, suggesting a possibility of applying them as predictive markers of response [68]. Similarly, another study showed that many genes that are responsible for erythroid differentiation are upregulated in erythroid progenitors of responders implying their capacity to terminally differentiate and survive, while erythrocytes of non-responders are stuck in the proliferative state.

Furthermore, several proteins that include carbonic anhydrase 1 and peroxiredoxin 2 were differentially expressed among responders and non-responders, implying the importance of proteomics as a predictor of response to hydroxyurea treatment. Hydroxyurea has also shown to upregulate S100A8, which is an important protein in phosphorylation of p38. As activation of γ-globin in hydroxyurea is dependent on p38 pathway, the above observation is important in predicting the response to treatment [29]. Another study revealed high levels of miR-210 and miR-486-3p levels in responders of hydroxyurea in comparison to non-responders [49]. In summary, the differential response to hydroxyurea treatment observed in patients with haemoglobinopathies is likely to be due to the cumulative effects of these models.

### Clinical use of hydroxyurea in sickle cell disease

Hydroxyurea has been in use for SCD from the early 1980s and received FDA approval for its treatment in adults in late 1990s. Currently, it is recommended for all patients with SCD. However, the extent of its use depends on the region and the country. Recurrent episodes of ischemia due to vaso-occlusion by sickled erythrocytes is the hallmark of SCD. Vaso-occlusion leads to multiple clinical manifestations, including painful crises, acute chest syndrome, strokes, dactylitis and leg ulcers. Hydroxyurea has shown to increase HbF and reduce the incidence of vaso-occlusive events, pulmonary hypertension, hospitalisations and mortality of patients with SCD in many clinical trials [21].

A meta-analysis involving eight independent studies and 899 patients concluded that hydroxyurea reduces the frequency of pain crises and acute complications in patients with SCD. It was also beneficial in reducing the incidence of stroke in high risk patients [70]. Another clinical trial conducted on 299 SCD patients observed a significant reduction in the duration of hospital stay due to painful crises and requirement of opioids [71]. A large prospective phase II clinical trial (LaSHS) reported a drastic decline in the incidence of severe painful crises, acute chest syndrome and hospital admissions and improvement of transfusion requirement in patients treated with hydroxyurea [61]. This study also reported significantly higher 10-year survival following hydroxyurea treatment. Additionally, several clinical trials that evaluate the efficacy of hydroxyurea on the paediatric population (NCT03789591), stroke prevention (NCT03948867) and neurological complications (NCT02556099) of SCD are underway.

The beneficial effects of hydroxyurea are extended to SCD variants like haemoglobin SC disease and sickle β-thalassaemia as well. A multicentre study conducted on 133 patients with haemoglobin SC disease revealed that hydroxyurea maintained a stable haemoglobin level and decreased the frequency of painful crises thus reducing the rate of hospitalization by 47% [39]. Another trial performed on patients with sickle β-thalassaemia showed improvement in haemoglobin, MCV and RBC morphology following hydroxyurea treatment [72]. Similarly, patients with sickle β-thalassaemia and SCA demonstrated comparable HbF responses of 20% and 19% respectively to hydroxyurea [73].

Although the primary mechanism of action of hydroxyurea is the induction of γ-globin and HbF, the effects of hydroxyurea in SCD is through multiple mechanisms. These include reducing the polymerisation of sickled RBC and endothelial activation state [74]. Additionally, cell adhesion to vascular endothelium is inhibited by hydroxyurea through downregulating cell adhesive molecules on reticulocytes (VLA 4 integrin and CD36) and mature RBCs (CD47) and limiting cell–cell and cell–matrix adhesions [62, 75]. Reduction of adhesion of cells to vascular endothelium is also achieved via reduction of white blood cells and platelets. The beneficial effect of hydroxyurea on pulmonary hypertension is through induction of NO/cGMP signalling and reduction of intravascular haemolysis. Improvement of haemoglobin concentration, oxygen saturation and anaemia and prevention of vaso-occlusive events also contribute to reduce pulmonary hypertension [76, 77].

### Clinical use of hydroxyurea in non-transfusion dependent β-thalassaemia

Patients in the less severe end of the clinical spectrum of β-thalassaemia syndromes have non-transfusion dependent (NTD) β-thalassaemia. These include β-thalassaemia intermedia and mild-moderate haemoglobin E β-thalassaemia [78, 79]. These patients do not require regular transfusions and are not transfusion dependent. However, they have a variable degree of chronic anaemia and features of extramedullary haematopoiesis, therefore, require infrequent RBC transfusions. Depending on the degree of anaemia and clinical severity, these patients have variable clinical features, including splenomegaly, leg ulcers, pulmonary hypertension, and organ dysfunction due to iron overload.

Hydroxyurea has shown promise in improving the haemoglobin levels and disease-associated complications in patients with NTD β-thalassaemia [80–82]. Many studies, including two meta-analyses involving 709 patients and 344 patients with NTD β-thalassaemia showed a significant reduction in transfusion requirement following hydroxyurea treatment [81] [83]. Nonetheless, the complete response rate in the two studies were 42% and 53% while partial response rates were 79% in both. These results indicate that a significant proportion of patients with NTD β-thalassaemia does not show any response to hydroxyurea treatment (commonly labelled as non-responders). Similarly, some studies have not been able to show a reduction in transfusion requirement among NTD β-thalassaemia patients despite having an increment of HbF [84]. The possible explanations for this difference in response were discussed previously.

In addition to decreasing the transfusion requirement, hydroxyurea has shown to be effective in mitigating extramedullary haematopoiesis and ineffective erythropoiesis in patients with NTD β-thalassaemia [85–87]. Treatment with hydroxyurea is associated with a significant reduction of spleen size in several studies [24, 26]. A study among a large cohort of patients with NDT β-thalassaemia revealed that hydroxyurea reduces the risk of leg ulcers, pulmonary hypertension and osteoporosis [88]. Furthermore, hydroxyurea was useful in treating paravertebral pseudotumor caused by extramedullary haematopoiesis in several case reports. Despite these beneficial effects, several aspects of hydroxyurea treatment in NTD β-thalassaemia that include optimal dosing and safety in children require further evaluation.

### Clinical use of hydroxyurea in transfusion dependent β-thalassaemia

Transfusion dependent (TD) β-thalassaemia is the most severe forms of β-thalassaemia, which encompasses β-thalassemia major and severe haemoglobin E β-thalassaemia [89]. These patients, except for a minority who were cured by allogeneic haematopoietic stem cell transplantation or gene therapy, require regular 2–5 weekly RBC transfusions and iron chelation life-long [90, 91]

Hydroxyurea has been used in many observational studies among these patients. A meta-analysis of eleven observational studies involving 859 patients with TD β-thalassaemia major concluded that hydroxyurea achieved a complete and partial response rate of 26% and 60% respectively [89]. However, two recent Cochrane reviews that analysed the effects of hydroxyurea in patients with TD β-thalassaemia concluded that the available evidence from clinical trials is insufficient to show hydroxyurea is effective in this group of patients [84, 92].

Due to the scarcity of data on the role of hydroxyurea in TD β-thalassaemia, many clinical trials are underway. These include a phase II randomised trial to assess the efficacy and safety of hydroxyurea in patients with β-thalassemia major (NCT03183375) and a phase III trial to evaluate the efficacy of hydroxyurea in improving oxidative stress and iron chelation in patients with β-thalassemia major (NCT04292314). We are currently conducting the first-ever randomised, double-blind placebo-controlled clinical trial to assess the efficacy and safety of hydroxyurea in patients with transfusion dependent β-thalassaemia (SLCTR/2018/024) [3].

### Safety profile of hydroxyurea

Hydroxyurea is a well-tolerated oral drug that has been in use for several decades. The most common adverse effect of hydroxyurea is cytopenia (approximately 20%) due to a dose-dependent and transient suppression of the bone marrow. Although it affects all haematological cell lineages, neutrophils are most commonly affected, resulting in mild to moderate neutropenia. Reticulocytopenia and thrombocytopenia are also reported. These haematological toxicities are reversible by withholding the drug for a few weeks or decreasing the dose [1, 37]. Another commonly reported side effect is hyperpigmentation of nails and skin, especially in palms and soles. Additionally, hydroxyurea is also known to cause headache and gastrointestinal symptoms that include nausea, vomiting, abdominal pain and constipation [21].

Other main concerns regarding the use of hydroxyurea are male infertility and increased risk of malignancies. Few clinical studies identified a negative effect on spermatogenesis in males [93, 94] while animal studies reported testicular toxicity in adult mice after subjecting to repeated doses of hydroxyurea [95, 96]. Although earlier studies raised concerns on the risk of developing leukaemia with long term exposure, patients who were on hydroxyurea for 10–20 years have not shown any increment in the incidence of cancers [1, 97]. Similarly, there is no convincing data that hydroxyurea is teratogenic. However, until satisfactory evidence is available, the prescription of hydroxyurea is discouraged during pregnancy and lactation [98–100].

### Use of hydroxyurea during COVID 19 pandemic

The COVID-19 pandemic which erupted in early 2020 disrupted the lives of many including patients with haemoglobinopathies who were on various transfusion regimens. The pandemic primarily affected the availability and delivery of blood for patients who were transfusion dependent. There was a severe sparsity in blood donors and health care personnel to collect, process, and deliver the blood. Most blood donation programmes were halted, and a severe shortage of blood was experienced across the globe, limiting the availability of blood for patients who were on regular transfusion regimens. For example, approximately 4000 blood distributions and over 130,000 blood donations were cancelled in the United States over a few weeks declining its blood reserve by 80% [101, 102].

Another problem encountered during the pandemic by patients with chronic diseases like SCD and thalassaemia is limited access to medical care. Strict social distancing and lockdown measures prevented patients from attending routine clinic appointments. Patients living in developing countries faced greatest difficulties; ironically, most patients with SCD and thalassaemia live in these regions. A recent study showed that access to healthcare for non-COVID related illnesses was significanlty lower in poorer countries including Bangaladesh, Kenya, Nigeria and Pakistan when compared to pre COVID-19 era [103].

Due to these obstacles, many patients with haemoglobinopathies received fewer than the usual number of transfusions. Caring physicians were compelled to look for and use alternative therapies for blood transfusions. Activin IIB receptor ligand trap luspatercept was recently approved as an adjunct to transfusions in patients with thalassaemia. In a recent phase 3 clinical trial in 336 patients with thalassaemia, subcutaneous luspatercept showed a 33% reduction in transfusion burden in patients with thalassaemia [104]. However, luspatercept is extremely costly and is not available in many developing countries where haemoglobinopathies are prevalent.

Therefore, as an immediate solution to the shortage of blood and limited access to medical care DeBaun recently proposed commencing hydroxyurea as an adjunct therapy for patients with SCD who were on regular transfusions [105]. Nickel and others endorsed this and soon released the preliminary results of their ongoing clinical trial to suggest escalated dose hydroxyurea treatment with transfusion as an approach to conserve blood during the pandemic [106]. Also, the recent position statement by the Thalassaemia International Federation on COVID-19 recommended the use of hydroxyurea in patients with thalassaemia to optimise blood use [107]. Thus it is clear that hydroxyurea could play a remarkable role by reducing transfusion requirements in patients with haemoglobinopathies, especially when donor blood is a limited resource.

## Conclusions

Hydroxyurea is a well-tolerated oral drug which has been in use for many decades. Through its actions of reversible inhibition of rNDP enzyme and fetal haemoglobin induction, it exerts many favourable effects on haemoglobin content, RBC indices, ineffective erythropoiesis and blood rheology in patients with β-haemoglobinopathies. It is currently approved for the treatment of SCD and is being widely used to treat NTD β-thalassaemia. Also, there are various observations to suggest that hydroxyurea is an important adjunct for the treatment of TD β-thalassaemia which should be confirmed by randomised clinical trials.

## Data Availability

Data sharing is not applicable to this article as no datasets were generated or analysed during the current study.

## Abbreviations

DNMT1: DNA methyltransferase 1
Hb: Haemoglobin
LDH: Lactate dehydrogenase
MCH: Mean corpuscular haemoglobin
MCV: Mean corpuscular volume
miRNA: MicroRNA
NO: Nitric oxide
NTD: Non transfusion dependent
RBC: Red blood cell
rNDP: Ribonucleoside diphosphate reductase
SCD: Sickle cell disease
TD: Transfusion dependent
